# Beauty in abstract paintings: perceptual contrast and statistical properties

**DOI:** 10.3389/fnhum.2014.00161

**Published:** 2014-03-21

**Authors:** Birgit Mallon, Christoph Redies, Gregor U. Hayn-Leichsenring

**Affiliations:** Experimental Aesthetics Group, Institute of Anatomy I, Jena University Hospital, University of Jena School of MedicineJena, Germany

**Keywords:** experimental aesthetics, perceptual contrast, abstract art, beauty, digital image analysis, self-similarity, color

## Abstract

In this study, we combined the behavioral and objective approach in the field of empirical aesthetics. First, we studied the perception of beauty by investigating shifts in evaluation on perceived beauty of abstract artworks (Experiment 1). Because the participants showed heterogeneous individual preferences for the paintings, we divided them into seven clusters for the test. The experiment revealed a clear pattern of perceptual contrast. The perceived beauty of abstract paintings increased after exposure to paintings that were rated as less beautiful, and it decreased after exposure to paintings that were rated as more beautiful. Next, we searched for correlations of beauty ratings and perceptual contrast with statistical properties of abstract artworks (Experiment 2). The participants showed significant preferences for particular image properties. These preferences differed between the clusters of participants. Strikingly, next to color measures like hue, saturation, value and lightness, the recently described Pyramid of Histograms of Orientation Gradients (PHOG) self-similarity value seems to be a predictor for aesthetic appreciation of abstract artworks. We speculate that the shift in evaluation in Experiment 1 was, at least in part, based on low-level adaptation to some of the statistical image properties analyzed in Experiment 2. In conclusion, our findings demonstrate that the perception of beauty in abstract artworks is altered after exposure to beautiful or non-beautiful images and correlates with particular image properties, especially color measures and self-similarity.

## Introduction

The field of experimental aesthetics attracted renewed interest in recent years. Two main approaches have emerged in this field. In one approach, the physiological and behavioral reactions of persons who view artworks are investigated. For example, in imaging studies, brain regions that are differentially activated by aesthetic visual stimuli were identified. Some of these regions belong to the self-reflective and reward systems of the brain (O'Doherty et al., 2003; Cela-Conde et al., 2004; Kawabata and Zeki, 2004). Other regions have previously been associated with moral judgment (Zaidel and Nadal, 2011; Avram et al., 2013) or are part of the default mode network (Vessel et al., 2012). At the behavioral level, researchers ask how persons perceive artworks or other visually pleasing stimuli in psychological experiments.

In the other approach, statistical features that characterize visually pleasing stimuli are analyzed by modern computational methods (reviewed in Hoenig, 2005; Graham and Redies, 2010). A pioneer in this field was Gustav Theodor Fechner. His hypothesis of the golden section, which he advanced in his influential book “Vorschule der Ästhetik” (Fechner, 1876), was one of the first attempts to directly measure image properties that relate to the aesthetic quality of images. Although later studies did not confirm any significant correlation between beauty ratings and particular geometric measures (for example, see McManus, 1980), Fechner set the foundations for a new scientific approach, i.e., the systematic search for stimulus properties that are associated with beauty. Nowadays, image analysis is based on firmly established empirical methods rather than on vague intuitive grounds. For example, computer-assisted algorithms are used to extract image features that characterize aesthetic images (Datta, 2006; Graham and Field, 2007; Redies et al., 2007a, 2012; Amirshahi et al., 2012), to predict emotional responses to paintings (e.g., Yanulevskaya et al., 2012) or to categorize painting styles (Wallraven et al., 2009). It is hoped that, in combination, the two approaches of experimental aesthetics will help us to understand what the specific properties of aesthetic images are and how they elicit brain responses that correlate with aesthetic judgments by the observers (Redies, 2007).

The appreciation of beauty in artworks depends to a large extent on cultural norms and previous exposure to art objects (Leder et al., 2004). Several studies suggest that people can differ considerably in their individual judgments (e.g., Jacobsen, 2004), but few studies of aesthetic responses have taken into account the individual differences between observers in their experimental design. Because of the large interindividual differences, a recent study by Vessel et al. (2012) used artworks as stimuli that were individually selected to correspond to each participant's strong personal preference. Interestingly, several studies showed that the personal aesthetic preferences of patients who suffer from dementia are relatively stable, even at late stages of the disease (Halpern et al., 2008; Graham et al., 2013; Halpern and O'Connor, 2013).

In the present study, we focused on the aesthetic perception of abstract art. Because the definition of beauty is a highly disputed matter, especially with respect to abstract artworks, we left it to the participants' assessment what they perceived as beautiful or not. To take into account individual aesthetic preferences, the 50 participants in our study first evaluated the beauty of 150 high-resolution abstract paintings and were then clustered into seven groups, each comprising participants with a preference for similar paintings.

Following this initial beauty evaluation, we carried out two experiments. In Experiment 1, we studied perceptual contrast with respect to beauty by adapting participants to cluster-specific subsets of their preferred and non-preferred paintings, respectively. Perceptual contrast is defined as a shift of the evaluation of a stimulus away from the evaluation of the preceding stimulus (e.g., see Baccus and Meister, 2004). Because individuals differ in their taste, we assumed that, by using individualized stimuli, contrast effects would be stronger than for images that were generally preferred or disliked by all participants. In Experiment 2, we studied the preferred paintings with respect to color and higher-order statistical image properties that have previously been linked to aesthetic perception and correlated these properties with the individualized evaluation data.

One possible explanation for perceptual contrast is visual adaptation. As Webster (2001) pointed out, adaptation processes are nothing exceptional and have been known for a long time (e.g., see Gibson and Radner, 1937). Adaptation is necessary because our environment is always changing and thus cannot be analyzed optimally by a visual system with fixed properties (Webster, 2001). The first scientific studies on adaptation dealt with relatively simple image features, such as the color aftereffect or the tilt aftereffect. The discovery of long-lasting aftereffects lead vision scientists to realize that the physiological correlate of adaptation must be more than simple fatigue of neural mechanisms or inhibitory mechanisms, as it had been assumed before (for a review, see Thompson and Burr, 2009). Recent research targeted relatively elaborate stimulus features in adaptation studies. For example, human faces have become widely studied stimuli, because they are of exceptional interest for human behavior. To name just a few of the results in this field, researchers demonstrated adaptation to gender (Troje et al., 2006), age (Schweinberger et al., 2010), and attractiveness (Rhodes et al., 2003).

However, the existence of a visual adaptation is not the only possibility to explain perceptual contrast. An altered evaluation of the stimulus after exposure to a differing stimulus might also be the result of a criterion shift. Criterion shifts can be described as changes of the central tendencies of the participants' individual psychometric functions (Morgan et al., 2012). The shift in evaluation can therefore not be taken as evidence for genuinely perceptual biases.

In a recent study, Hayn-Leichsenring et al. (2013) demonstrated an attractiveness aftereffect for face photographs and art portraits. In the case of art portraits, similar aftereffects were detected for beauty. In their study, attractiveness was defined as the physical allurement of a face whereas beauty was considered as a more general property of images and referred to the pleasure derived from the composition of the image (or artwork). Following this definition, the authors found a strong correlation of the beauty ratings with the attractiveness ratings for art portraits, suggesting the possibility that participants may confound the two features easily.

In order to confirm and extend these findings, the goal of Experiment 1 in the present study was to explore whether perceptual contrast for the perception of beauty can be demonstrated even in the absence of semantic content that can potentially confound the assessment of beauty. The existence of an aftereffect on beauty might possibly have an evolutionary advantage. The ability to adjust one's perception to the currently relevant range of beauty in the environment can be a critical benefit, as it improves the differential appraisal of actual stimuli. There are indeed hints that a long-term adaptation to aesthetic features exists (Carbon, 2011). To our knowledge, however, short-term aftereffects on artworks have been barely investigated to date. The only publication on this topic was restricted to a single style of painting (Carbon et al., 2007).

In the present experiment, we studied contrast effects on abstract images that do not depict recognizable objects. Although we cannot exclude that participants projected some imaginary content into the paintings, the influence of individual preferences for depicted objects or scenes is minimized by using abstract paintings so that beauty (as a formal property of the paintings) can be rated relatively independent of a preference for the semantic content of the paintings.

In Experiment 2, we focused on statistical image features that were previously analyzed in studies of aesthetic images. The image features were calculated for the abstract artworks that were rated by the participants in Experiment 1. The resulting values were correlated with beauty ratings in order to challenge their usefulness for predicting individual aesthetic preferences. Additionally, we investigated the relation of the physical image properties to the adaptation and the clustering of the participants. In the following sections, the image features studied will be introduced briefly.

### Self-similarity

The property of self-similarity implies that an object as a whole has an appearance similar to its parts. Closely related concepts are scale-invariance and fractality (Taylor et al., 2011). For example, subsets of aesthetic monochrome artworks possess a scale-invariant Fourier spectrum, which means that the relative strength of coarse and fine structures changes little as one zooms in and out of the image (Graham and Field, 2007; Redies et al., 2007b; Taylor et al., 2011; Melmer et al., 2013). Amirshahi et al. (2012) studied self-similarity in images of artworks directly by using a modern computational approach, the Pyramid of Histograms of Orientation Gradients (PHOG) method (Bosch et al., 2007).

### Complexity

Berlyne (1974) related complexity to factors such as the regularity of the pattern, the amount of elements that form the scene, their heterogeneity, or the irregularity of the forms. Berlyne's idea that a high aesthetic appeal goes along with an intermediate level of complexity is still considered valid today (for a review, see Nadal, 2007), although the range of complexity values observed in artworks is rather wide (Redies et al., 2012).

### Anisotropy

According to Koch et al. (2010), large subsets of Western artworks tend to possess a more isotropic Fourier spectrum than their corresponding real-world models. Similar findings were obtained by Redies et al. (2012), who described that overall gradient strength is more uniformly distributed across orientations in large subsets of colored artworks of Western provenance, compared to other categories of images. The perceptual significance of this finding remains unclear at present.

### Birkhoff-like measure

According to Birkhoff (1933), aesthetic value depends on the ratio of order and complexity. Following this idea, we substituted order by self-similarity to obtain a Birkhoff-like measure, as described in Redies et al. (2012).

### Aspect ratio

Modern research has shown no evidence that a certain aspect ratio might be preferred over others, but points out the need for measurements on different types of images to answer this question more comprehensively (McManus, 1980; Russell, 2000). In the present study, we therefore correlated the beauty ratings with the aspect ratios of the abstract paintings.

### Color measures

The influence of color on aesthetic appreciation has been emphasized before, particularly in approaches that use computational methods for quantifying the aesthetic quality of photographs and paintings (Li and Chen, 2009; Marchesotti et al., 2011). Forsythe et al. (2011) stressed the importance of color as a medium that artists use to communicate with the observer. Yanulevskaya et al. (2012) investigated emotional response patterns to certain colors. They revealed a correlation of bright colors and smooth lines with positive emotions, as opposed to dark colors and chaotic texture that go along with negative emotions. Palmer and Schloss (2010) explained such correlations with their ecological valence theory, stating that color preferences are associated with preferred objects of the same colors. For example, fresh fruits are mostly of bright color, while rotten food, and excrements are normally of a dark brownish color that is naturally averted by most people. To date, several different color measures have been applied to colored artworks and photographs (e.g., see Datta, 2006). In the present work, we calculated color measures in 3 different color spaces (RGB, HSV, and Lab) that have been used in aesthetic quality assessment of images previously.

Our study thus combines the two approaches in experimental aesthetics mentioned above (behavioral and computational). In Experiment 1, we investigate, at the behavioral level, how the aesthetic judgments change after the viewing of most beautiful and least beautiful images. In Experiment 2, we ask which objective statistical properties in the same set of aesthetic images correlate with the beauty judgments. In both experiments, individualized evaluations of beauty are explicitly incorporated into the experimental design.

## Methods

In Experiment 1, participants took part in two tests to investigate adaptation to images evaluated as most and least beautiful, respectively. In Experiment 1A, each participant rated the abstract images according to his or her own personal concept of beauty. In Experiment 1B, participants were exposed to paintings that they considered to be either of high or low beauty. Subsequently, participants rated some of the images used in Experiment 1A again. For these images, the initial ratings (Experiment 1A) and the ratings that were given in Experiment 1B were compared to detect perceptual contrast.

### Experiment 1A: beauty rating of the images

#### Participants

Fifty participants (19–44 years old, *M* = 22.7 years old, 13 males) attended this study. Most of them were students, in particular of medical sciences, but other fields of studies and professions were reported also. None of them had received professional training in the fine arts. All participants declared having normal or corrected-to-normal visual acuity and gave their written informed consent after receiving an explanation of the procedures. The study design complied with the ethical guidelines of the Declaration of Helsinki and was approved by the ethics committee of Jena University Hospital.

#### Stimuli

One hundred-fifty images of abstract paintings or drawings were scanned from different art books. We chose only abstract artworks, which did not carry any clear semantic content and did not depict any recognizable objects. Abstract artworks were selected to minimize the influence of a preference for image content on the evaluation of the images. The artworks are listed in the Appendix and were from a variety of abstract painters of the 20th and 21st century and from different cultural backgrounds of the Western hemisphere. A maximum of six artworks was included from each artist in order to decrease the influence of any preference or aversion for a given painter on the results. An effort was made to select artworks from art books as randomly as possible, regardless of personal preference by the authors.

Digitization of the images was carried out with a commercial color scanner (Perfection 3200 Photo, Seiko Epson Corporation, Nagano, Japan) in RGB color format. Care was taken that the images scanned were of high quality and did not contain obvious artifacts like paper folds or stains. Moreover, only pictures of a size that enabled high-quality scans were chosen. No image enhancement algorithms were applied. All pictures were reduced in size to 1024 pixels on the longest side by isotropic bicubic interpolation for display on the screen, on which stimuli were presented at a size of 165 mm (10.5° of visual angle).

#### Procedure

Images of all artworks were shown separately and in a random sequence on a black screen (Color Edge CG241W LCD monitor, EIZO Europe, Germany). A chin rest assured a constant viewing distance of 90 cm. The participants were asked to rate the artworks on a scale from 1 (most beautiful) to 4 (least beautiful), which reflected the grading scheme in the German school system. In the course of the trial, every participant had to evaluate each picture once.

**Figure 1:**
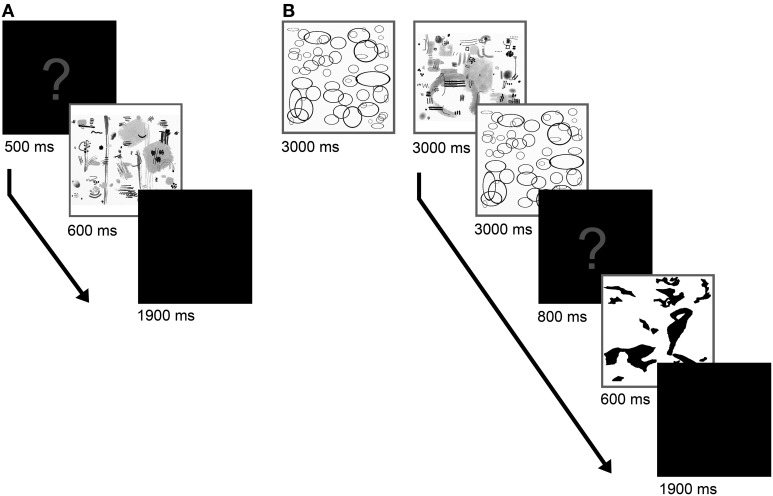
**Schedule for Experiment 1A (A) and Experiment 1B (B)**. In the second part of the experiment, an adaptation phase preceded the evaluation phase and the adaptation was reconditioned by presentation of two adaptor images before the display of each test stimulus.

The experiment was performed using the MATLAB program (version R2008A). The schedule of Experiment 1A is depicted in Figure 1A. Prior to presenting each image, a question mark was displayed (500 ms), followed by the image itself (600 ms) and a period of 1900 ms, during which a black screen was displayed and the participants were asked to rate the beauty of the pictures by pressing one of four keys labeled “1” to “4.” We used a relatively short time period of 600 ms (see also Hayn-Leichsenring et al., 2013) because this study focuses on perceptual rather than on cognitive effects. Moreover, the relatively short presentation times decreased the likelihood that participants perceived spurious content in the abstract images or projected imaginary content into them. After every 30 images, the participants were allowed to take a short break.

### Experiment 1B: perceptual contrast on most and least beautiful images

#### Participants

Forty-two participants (19–44 years old, *M* = 22.7 years old, 9 males), who had attended Experiment 1A about 5 weeks before, took part in this trial.

As the evaluation on beauty was quite heterogeneous among the participants, i.e., groups of participants showed a specific “taste” in their evaluation, we chose to perform the experiment (Experiment 1B) with seven clusters. To create these clusters, the data of all participants were subclassified with the k-means-clustering method according to their individual ratings on beauty. Clustering allowed allocation of participants to subgroups that resembled each other in their individual preference. The clusters were created for two purposes. Firstly, we used them to carry out the experiment on perceptual aftereffects (Experiment 1B) with paintings that were preferred or non-preferred by small groups of participants. We expected that effects would be larger if the adaptors closely corresponded to the beauty preferences of each participant. Secondly, the formation of clusters allowed us to search for correlations between image properties and cluster-specific ratings and to ask whether individual patterns of beauty preferences were associated with particular image properties (Experiment 2). A statistical analysis of the sum of squares (within clusters), the Bayesian information criterion and Dunn's index did not provide any consistent indication of which number of clusters (between 2 and 10) would be optimal. Therefore, we chose to carry out the experiment with seven clusters and a minimum number of 4 participants in each cluster. A larger numbers of clusters would have resulted in exceedingly small numbers of participants (<4) in each cluster.

The seven clusters used for Experiment 1B consisted of ten participants (1 cluster), nine participants (1 cluster), seven participants (3 clusters), six participants (1 cluster), and four participants (1 cluster), respectively. For the statistical analysis, we divided participants into 3–7 clusters in order to investigate the stability of the clusters and their statistical properties as the number of clusters increases.

#### Stimuli

**Figure 2:**
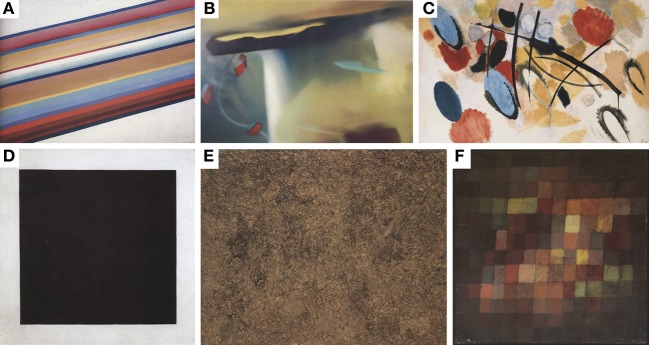
**Examples of paintings that were rated as most beautiful (A–C) and least beautiful (D–F), respectively**. The paintings are **(A)** “Movement in Space” by Michail Matjuschin (1917/18). **(B)** Abstract painting by Gerhard Richter [1977. (c) Gerhard Richter Images, Köln, 2013]. **(C)** “Mit aphoristischem Rot” by Ernst Wilhelm Nay [1954. (c) Succession Nay, VG Bild-Kunst, Bonn, 2013]. **(D)** “Black Square” by Kasimir Malewitsch (1923). **(E)** “Joie de terre” by Jean Dubuffet (1959; Succession Dubuffet, VG Bild-Kunst, Bonn, 2013); and **(F)** “Alter Klang” by Paul Klee (1925).

For the adaptation phase, we chose the 15 images that were rated to be the most beautiful and the least beautiful, respectively, within each cluster. Thus, every participant adapted on a set of artworks that came close to his individual assessment of what are the most and least beautiful images. Examples of the artworks with generally high and low ratings are shown in Figure 2.

In the evaluation phase, we used the 60 images that had received an average rating for their particular cluster in Experiment 1A. Images of the evaluation set were not part of the assortment used for adaptation. Size and manner of the presentation of the images during the adaptation phase were the same as in Experiment 1A. The pictures used for evaluation had a reduced size of 115 mm on the longest side (7.3° of visual angle, 720 pixels on the screen). We resized the images to investigate adaptive coding that is not exclusively a property of early stages of visual processing (Clifford et al., 2007).

#### Procedure

As depicted in Figure 1B, the experimental trial consisted of an adaptation phase followed by an evaluation phase. For adaptation, the participants were asked to look at the 15 images that were evaluated to be the most beautiful (and in a second experimental block the least beautiful) in their respective cluster. The images were shown consecutively and in a repeating manner. Images were shown for 3000 ms three times each, so that the entire phase lasted 2 min and 15 s. In the subsequent evaluation phase, the adaptation was reconditioned before the presentation of each target image by showing two images that were randomly selected from the 15 images considered most beautiful (2 × 3000 ms; or least beautiful, respectively). After reconditioning, a question mark appeared (800 ms) followed by the target image (600 ms). Then, a black screen was presented for 1900 ms, during which the participants were asked to respond as described in Experiment 1A. In order to prevent a bias that is caused by the sequence of the adaptors, half of the participants adapted first on most the beautiful images and half on the least beautiful ones. In both experimental blocks of Experiment 1B (adaptation on most beautiful and least beautiful images), the participants evaluated all the 60 images, which had previously received an average rating (see above).

### Experiment 2: image analysis

As mentioned for Experiment 1B (section Participants), we obtained 3–7 clusters of the participants with respect to their evaluation results of the 150 color images of abstract paintings (Experiment 1A) using the k-means method. This corresponded to a total of 25 different clusters (i.e., cluster 1/3, 2/3… 7/7). The aim of Experiment 2 was to identify correlations between the beauty ratings and perceptual contrast effects, respectively, with a variety of statistical image properties that were previously associated with aesthetic stimuli (see Introduction).

For the statistical analysis, all images were down-sampled to a uniform size of 100,000 pixels because some of the scanned images contained halftone dots that were visible at higher resolutions. This artifact would have affected the calculation of self-similarity (section Self-similarity) in particular.

Self-similarity, complexity, anisotropy, and the Birkhoff-like measure were calculated using the PHOG method, a computational method that was originally developed for object classification in images (Bosch et al., 2007). This method was used to measure statistical properties of photographs and artworks before (Amirshahi et al., 2012; Redies et al., 2012). The analysis was carried out using MATLAB 2008A. We recently described the method in detail in the appendix to the open-access publication by Braun et al. (2013); see also (Redies and Groß, 2013).

In brief, the method is based on a pyramid approach: Firstly, the HOG feature (Dalal and Triggs, 2005) for the entire image is calculated at the ground level (level 0). The HOG feature represents the histogram of the mean strength of the luminance gradients binned in 16 equally sized orientation bins that cover the full 360° of orientations in the image. In the second step, the image was divided into 4 rectangles of the same size and the HOG features were calculated again for each rectangle (level 1). Each of the 4 subimages was again divided into equal rectangles and the HOG features were calculated for the resulting 16 subimages as well (level 2). We took this approach up to level 3. Within each HOG feature, the strengths of the binned gradients were normalized. For the analysis of the color images, the images were converted to the Lab color space. For each pixel in the color image, the maximum gradient magnitude in the L, a and b color channels was used for the HOG calculation (gradient image).

#### Self-similarity

For calculating self-similarity, we compared the HOG features of the subimages on the third level with the HOG features of the entire image on level 0 using the Histogram Intersection Kernel (Barla et al., 2002). The third level proved to deliver the most reliable results in previous work because the differences between the subimages are more significant than at the lower level and yet robust and reliable (Amirshahi et al., 2012; Redies and Groß, 2013).

#### Complexity

In the gradient image, the sum of the strengths of all oriented gradients, which correspond to edges or lines with different orientations in the image, was used as a measure of complexity (Redies et al., 2012). This measure is highly correlated (Braun et al., 2013) with another measure of complexity, the fractal dimension (Mureika and Taylor, 2013).

#### Anisotropy

The standard deviation of the luminance gradient strengths in the 16 orientation bins at level 3 served as a measure of anisotropy. This property describes in how far the distribution of the oriented gradient strengths across all orientations deviates from a uniform distribution. A value close to zero indicates a uniform distribution of the orientation gradients across orientations.

#### Birkhoff-like measure

A Birkhoff-like measure was defined as the quotient of self-similarity over complexity, as introduced by Redies et al. (2012).

#### Aspect ratio

This measure is the quotient of height and width of an image.

#### Color measures (HSV, RGB, and Lab color channels)

Color features were analyzed in three different color spaces (HSV, RGB, and Lab) with Matlab and ImageJ (Abramoff et al., 2004). The original RGB-coded images were converted into the HSV color space with the MATLAB program and into the Lab color space with the Photoshop program (Adobe, Mountainview, CA). Subsequently, the strength (average pixel value) of each color channel was calculated.

## Results

### Beauty rating of the images (experiment 1A)

**Figure 3:**
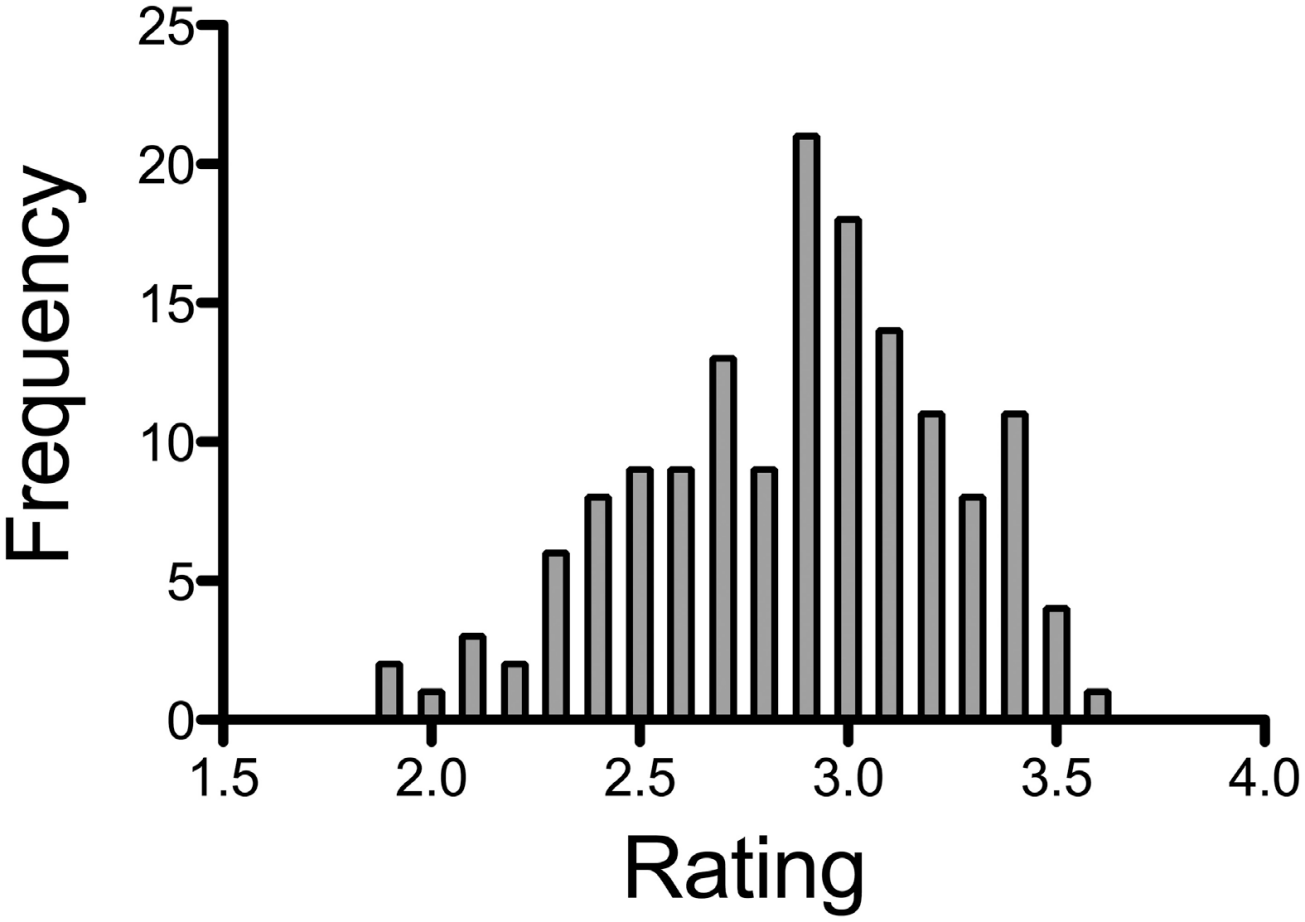
**Results of the rating experiment (Experiment 1A)**. Frequency represents the number of images that received the respective average rating.

For each image, the mean beauty rating was calculated for all participants. The mean rating ranged from 1.92 (for the most positively rated, i.e., “most beautiful”) to 3.6 (for the most negatively rated, i.e., “least beautiful”) [Mean (*M*) = 2.88, *SD* = 0.37]. Examples of paintings that received generally high and low beauty ratings, respectively, are shown in Figure 2. The distribution of the ratings for all paintings is shown in Figure 3. The average rating scores of individual participants ranged from 1.97 to 3.58. We used the results of Experiment 1A to cluster the participants into 7 subgroups (see section Participants).

### Perceptual contrast on most and least beautiful images (experiment 1B)

**Figure 4:**
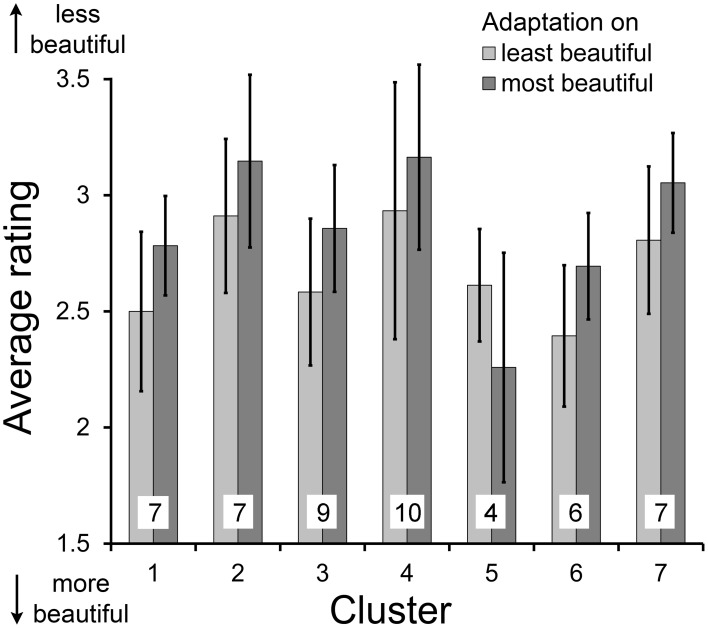
**Average ratings on beauty of abstract images after adaptation to most beautiful and least beautiful stimuli in Experiment 1 for each cluster (numbered 1–7)**. Error bars indicate standard deviation. All differences between adaptations to most beautiful and least beautiful stimuli were significant (*p* < 0.01). The numbers at the bottom of the columns indicate the number of participants in each cluster.

In this experiment, we obtained two ratings from each participant for each image: one after exposure to most beautiful images and one after exposure to least beautiful images. The average rating was 2.84 (*SD* = 0.19) after exposure to beautiful images and 2.68 (*SD* = 0.20) after exposure to least beautiful images, respectively. In all clusters (with the exception of cluster 5), images were rated as more beautiful after exposure to least beautiful stimuli, when compared to the ratings after exposure to most beautiful images. A paired *t*-test across images confirmed the significance of this perceptual contrast effect (*R* = 0.808; *df* = 131; *t* = −10.468; *p* < 0.001). Results for all seven clusters are shown in Figure 4.

### Statistical image analysis (experiment 2)

**Figure 5:**
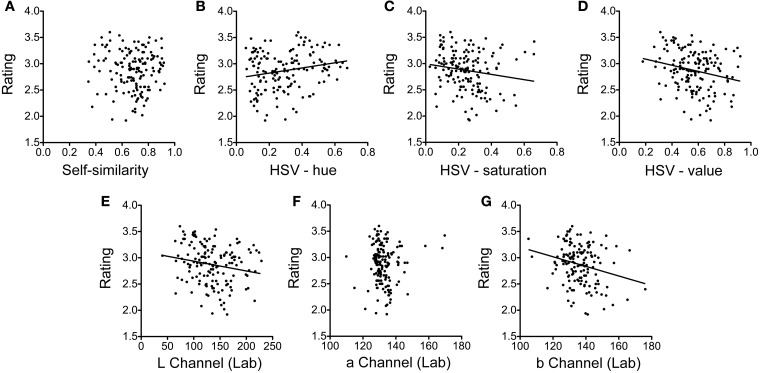
**Dot plots of the average beauty rating plotted as a function of self-similarity (A), color hue (B), color saturation (C), and color value of the HSV color space (D), and the three channels of the Lab color space (E–G)**. Each dot represents one of 150 images rated in Experiment 1A. The lines represent regression lines for the significant correlations only (**B–E**,**G)**. For self-similarity and the red-over-green (a) channel of the Lab space **(F)** correlations were found for individual clusters (see Figure 6), but not for the group of all participants shown in this figure.

**Figure 6:**
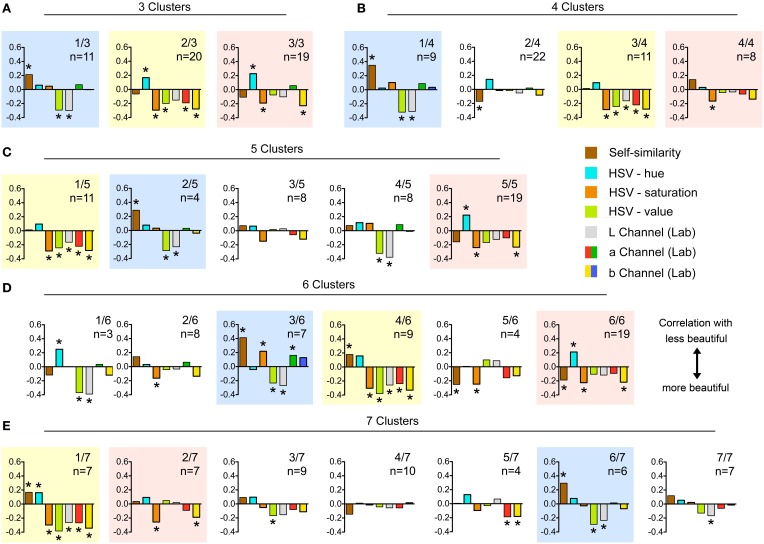
**Spearman coefficients for the correlations between image properties and beauty ratings**. For the color coding of the different image properties, see the explanation on the right hand side of **(C)**. **(A–E)** show the results for the different numbers of clusters (3–7 clusters, respectively). The colored backgrounds highlight clusters of similar preferences, independent of how many clusters (3–7) were formed. ^*^Indicates significant correlations (*p* < 0.05 or lower).

Over all participants (without clustering), we found significant correlations of beauty ratings with the following color measures: the hue channel (Spearman coefficient ρ = 0.182, *p* < 0.05; Figure 5B), the saturation channel (ρ = −0.217, *p* < 0.01; Figure 5C), and the value channel (ρ = −0.277, *p* < 0.01; Figure 5D) in the HSV space; the green color channel in the RGB color space (Spearman ρ = −0.217, *p* < 0.01); and the luminance (L) channel (ρ = −0.206, *p* < 0.05; Figure 5E) and the yellow-over-blue channel (b channel) in the Lab color space (ρ = −0.224, *p* < 0.01; Figure 5G). The other statistical measures, for example self-similarity (Figure 5A) and the red-over-green channel (a channel) in the Lab color space (Figure 5F), did not correlate with overall beauty ratings. We next analyzed the data for each cluster of participants separately. Results are listed in Table 1. The correlations between the most eminent statistical features and the beauty ratings are depicted in Figure 6 for each cluster.

**Table 1:** **Spearman coefficients *ρ* for the correlations between beauty ratings and image properties**.

	**Self-similarity**	**Complexity**	**Anisotropy**	**Birkhoff-like measure**	**HSV color hue**	**HSV color saturation**	**HSV color value**	**RGB red**	**RGB green**	**RGB blue**	**Lab-L lightness**	**Lab-a (red-green)**	**Lab-b (yellow-blue)**
Cluster 1/3	0.212^**^	−0.112	0.174^*^	0.079	0.061	0.047	−0.294^**^	−0.253^**^	−0.308^**^	−0.262^**^	−0.301^**^	0.069	−0.001
Cluster 2/3	−0.065	−0.018	0.052	0.10	0.167^*^	−0.295^**^	−0.199^*^	−0.184^*^	−0.165^*^	−0.041	−0.152	−0.191^*^	−0.278^**^
Cluster 3/3	−0.107	0.048	0.055	0.155	0.228^**^	−0.191^*^	−0.075	−0.66	−0.108	0.03	−0.102	0.058	−0.229^**^
Cluster 1/4	0.349^**^	−0.111	0.131	0.067	0.023	0.102	−0.32^**^	−0.276^**^	−0.327^**^	−0.286^**^	−0.309^**^	0.088	0.034
Cluster 2/4	−0.17^*^	0.034	0.031	−0.012	0.143	−0.015	−0.018	0.046	−0.075	−0.015	−0.05	0.02	−0.081
Cluster 3/4	0.013	−0.036	0.038	0.061	0.095	−0.288^**^	−0.244^**^	−0.239^**^	−0.2^*^	−0.085	−0.163^*^	−0.221^**^	−0.281^**^
Cluster 4/4	0.141	0.09	0.00	0.178^*^	0.03	−0.164^*^	−0.042	−0.036	−0.022	0.081	−0.034	−0.064	−0.136
Cluster 1/5	0.013	−0.036	0.038	0.061	0.095	−0.288^**^	−0.244^**^	−0.239^**^	−0.2^**^	−0.085	−0.163^*^	−0.221^**^	−0.281^**^
Cluster 2/5	0.289^**^	−0.066	0.014	0.089	0.076	0.032	−0.287^**^	−0.25^**^	−0.277^**^	−0.213^**^	−0.232^**^	0.031	−0.038
Cluster 3/5	0.072	0.105	0.028	0.142	0.065	−0.149	0.017	0.022	0.04	0.131	0.028	−0.057	−0.12
Cluster 4/5	0.074	−0.117	0.188^*^	0.01	0.115	0.105	−0.322^**^	−0.288^**^	−0.342^**^	−0.304^**^	−0.378^**^	0.086	−0.008
Cluster 5/5	−0.158	−0.007	0.071	0.117	0.222^**^	−0.24^**^	−0.166	−0.099	−0.125	−0.001	−0.122	−0.101	−0.233^**^
Cluster 1/6	−0.118	−0.145	0.207^*^	0.039	0.25^**^	0.001	−0.37^**^	−0.325^**^	−0.34^**^	−0.26^**^	−0.39^**^	0.034	−0.12
Cluster 2/6	0.141	0.09	0.00	0.178^*^	0.03	−0.164^*^	−0.042	−0.036	−0.022	0.081	−0.034	0.064	−0.136
Cluster 3/6	0.413^**^	−0.077	0.107	0.04	−0.041	0.219^**^	−0.234^**^	−0.215^**^	−0.3^**^	−0.308^**^	−0.269^**^	0.161^*^	0.128
Cluster 4/6	0.179^*^	−0.061	−0.003	0.048	0.158	−0.303^**^	−0.38^**^	−0.358^**^	−0.291^**^	−0.152	−0.257^**^	−0.236^**^	−0.33^**^
Cluster 5/6	−0.251^**^	0.034	0.028	0.064	0.004	−0.247^**^	0.098	0.098	0.08	0.123	0.086	−0.157	−0.128
Cluster 6/6	−0.188^*^	−0.006	0.073	0.116	0.212^**^	−0.226^**^	−0.105	−0.087	−0.119	−0.001	−0.117	−0.093	−0.219^**^
Cluster 1/7	0.167^*^	−0.059	0.01	0.036	0.162^*^	−0.298^**^	−0.384^**^	−0.377^**^	−0.295^**^	−0.156	−0.264^**^	−0.267^**^	−0.343^**^
Cluster 2/7	0.035	0.077	−0.016	0.117^*^	0.093	−0.257^**^	0.048	−0.04	−0.018	0.081	0.019	−0.091	−0.19^*^
Cluster 3/7	0.092	−0.027	0.007	−0.061	0.096	−0.052	−0.168^*^	−0.184^**^	−0.17^*^	−0.11	−0.155	−0.08	−0.112
Cluster 4/7	−0.146	−0.119	−0.079	−0.017	0.009	−0.014	−0.042	−0.019	−0.078	−0.096	−0.055	−0.056	0.016
Cluster 5/7	0.006	0.00	−0.143	0.009	0.13	−0.096	−0.028	−0.064	0.036	0.072	0.067	−0.187^*^	−0.181^*^
Cluster 6/7	0.296^**^	−0.111	0.036	0.076	0.078	−0.027	−0.291^**^	−0.247^**^	−0.281^**^	−0.199^*^	−0.235^**^	0.015	−0.069
Cluster 7/7	0.119	−0.097	0.057	−0.017	0.054	0.023	−0.13	−0.131	0.117	−0.151	−0.167^*^	−0.061	−0.014
Average evaluation	−0.024	−0.032	0.085	0.116	0.18^*^	−0.217^**^	−0.227^**^	−0.199^*^	−0.217^**^	−0.097	−0.206^*^	−0.115	−0.224^**^

Significant correlations:

* *p < 0.05*.

** *p < 0.01*.

Although there was no correlation over all participants (Figure 5A), we found significant correlations (*p* < 0.05 or lower) for self-similarity with beauty ratings in 10 of 25 clusters. Correlations were in both directions. For example, in the analysis with 6 clusters (Figure 6D), we found 4 clusters with a significant correlation, of which 2 were positive and 2 negative (ρ = 0.413, *p* < 0.01; ρ = 0.179, *p* < 0.05; ρ = −0.251, *p* < 0.01 and ρ = −0.188, *p* < 0.05; Table 1). Of the other 6 significant correlations of self-similarity with beauty ratings (Figures 6A–C,E), 5 were positive and 1 negative. Note that a positive value implies that more self-similar images were rated as less beautiful.

Other correlations with beauty ratings were found for color measures (Table 1). In the HSV color space, hue showed correlations in 6 clusters, and both saturation and value in 13 of 25 clusters (Figure 6). The correlations of color saturation and color value with the beauty rating were nearly all positive with one exception in one cluster, in which a low saturation was preferred. In the RGB color space, we found highly significant correlations with red (13 clusters), green (13 clusters), and blue (7 clusters). Figure 6 shows the results from the Lab color space only because the results can be interpreted more easily in terms of preferences for specific colors. In the Lab color space, there were preferences for lightness (12 clusters), as well as for red over green (5 clusters), for green over red (1 cluster), and for yellow over blue (10 clusters).

According to these correlations, we can characterize the clusters as follows: Members of cluster 1/3, 1/4, 2/5, 3/6, and 6/7 (shadowed by blue color in Figure 6) tend to prefer bright images of a comparatively low self-similarity and a high color value. In addition, significant preference for highly saturated color and for green color shade over red ones can be found in cluster 3/6. In the group of clusters shadowed in by yellow in Figure 6 (clusters 2/3, 3/4, 1/5, 4/6, and 1/7), low self-similarity is a crucial factor for only 2 out of the 5 clusters. Furthermore, nearly all of these clusters show preference for bright and highly saturated images with a high color value, and they favor reddish over greenish colors as well as yellowish over bluish ones. The third cluster group (shadowed by red color in Figure 6) comprises the clusters 3/3, 4/4, 5/5, 6/6, and 2/7. All of these clusters show a significant preference for highly saturated colors and, with the exception of cluster 4/4, also for yellow over blue color shades. A preference for highly self-similar images was detected in clusters 2/4, 5/6, and 6/6.

We found no or only one single correlations for each of the other features (complexity, anisotropy, the Birkhoff-like measure and the aspect ratio; *p* < 0.05).

Color hue is a circular measure. Therefore, we performed an additional analysis after splitting the hue values into six groups, each reflecting one color range (red, yellow, green, cyan, blue, and magenta). We did not find a significant preference for a specific color in any of the clusters.

In addition to our analyses over participants, we performed two multivariate linear regression analyses across images to evaluate in how far the average beauty rating and the perceptual contrast were predictable based on the statistical image properties. In the first analysis, we considered the average beauty rating as dependent factor and the statistical image properties as independent factors. We found that higher values in the Lab-a channel lead participants to rate images as less beautiful (standardized β = 0.206, *p* < 0.05). For the Lab-b channel, higher values lead participants to rate images as more beautiful (standardized β = −0.284, *p* < 0.05). Overall, the analysis revealed a low predictability of the system (*R*^2^ = 0.134). In the second multivariate linear regression analysis, the perceptual contrast was considered as dependent factor, while the statistical image properties were used as independent factors. We found a significant positive effect of PHOG self-similarity on the perceptual contrast (standardized β = 0.203, *p* < 0.05). Overall, the analysis revealed a low predictability of the system (*R*^2^ = 0.084; see Table 2 for complete results).

**Table 2:** **Regression coefficients (standardized *β*) for the overall correlations between average beauty ratings and the magnitudes of the perceptual contrast, respectively, and selected image properties**.

	**Self-similarity**	**Complexity**	**Anisotropy**	**Birkhoff-like measure**	**HSV color hue**	**HSV color saturation**	**HSV color value**	**Aspect ratio**	**Lab-L lightness**	**Lab-a (red-green)**	**Lab-b (yellow-blue)**
Average beauty	−0.017	0.029	0.074	0.074	−0.007	0.150	−0.060	−0.020	−0.092	0.206^*^	−0.284^*^
Perceptual contrast	0.203^*^	−0.071	−0.164	0.045	0.059	0.001	0.079	0.047	−0.026	0.005	−0.105

* *Significant at p < 0.05*.

Furthermore, to investigate in how far the ratings by the participants from the different clusters were predictable based on the statistical image properties, the data were entered into a linear mixed model analysis over images, considering the allocation to the clusters as fixed factor, the respective mean beauty rating for each cluster as dependent factor, and the statistical properties of each image as covariates. We found that the effect on the subjective rating of images differed between clusters for the following image properties: Self-similarity (*F* = 10.23, *p* < 0.001), complexity (*F* = 2.29, *p* < 0.05), Birkhoff-like measure (*F* = 10.02, *p* < 0.001), color hue (*F* = 9.61, *p* < 0.001), color saturation (*F* = 3.68, *p* < 0.05), and the aspect ratio (*F* = 4.08, *p* < 0.05). Results for a detailed analysis of the differences between the clusters with regard to the interaction between statistical image properties and cluster membership are provided in Table 3.

**Table 3:** **Results for the linear mixed model analysis over images**.

	**Self-similarity**		**Complexity**		**Anisotropy**		**Birkhoff-like measure**		**HSV color hue**		**HSV color saturation**		**HSV color value**		**Aspect ratio**	
	***RC***	***p***	***RC***	***p***	***RC***	***p***	***RC***	***p***	***RC***	***p***	***RC***	***p***	***RC***	***p***	***RC***	***p***
Cluster 1	−0.345	0.308	−0.002	0.790	−154	0.378	−0.455	0.010	−0.664	0.029	1.329	<0.001	0.677	0.049	0.247	0.124
Cluster 2	−1.170	0.001	0.013	0.120	−282	0.107	−0.555	0.002	−1.425	<0.001	0.245	0.549	0.932	0.007	0.157	0.317
Cluster 3	0.022	0.946	−0.005	0.501	−319	0.069	−0.665	<0.001	−1.106	<0.001	0.827	0.045	0.214	0.529	0.200	0.208
Cluster 4	−1.603	0.000	0.007	0.401	−454	0.010	−0.291	0.086	−1.928	<0.001	0.876	0.033	0.691	0.044	0.655	<0.001
Cluster 5	−1.023	0.003	0.019	0.023	−436	0.014	−1.185	<0.001	−0.571	0.059	−0.243	0.552	0.783	0.023	0.301	0.065
Cluster 6	0.512	0.133	0.001	0.941	−299	0.089	−0.575	0.002	−1.456	0.000	0.729	0.076	0.345	0.312	0.491	0.005

*Displayed are the regression coefficients for the difference of the effect of the statistical image properties on subjective ratings between each cluster and the reference cluster (cluster 7). The regression coefficients reflect the interaction between statistical image properties and cluster membership. RC, regression coefficient; p, p value*.

## Discussion

In this study, we demonstrate an aftereffect for perceived beauty of abstract artworks (Experiment 1B). Participants rated the images as more beautiful after adaptation to least beautiful images and *vice versa*. Moreover, we correlated beauty ratings and the magnitude of the perceptual contrast with specific image properties that have been studied before in the context of aesthetic perception (Experiment 2). The abstract artworks used in the present study have the advantage that the effect of semantic content, which might affect the assessment of beauty (Experiment 1A), is minimized.

### Perceptual contrast

Aftereffects on artworks have been shown previously, but only for adaptation to a single style of painting (Carbon et al., 2007) and for adaptation to the beauty of portrait paintings (Hayn-Leichsenring et al., 2013). Therefore, to the best of our knowledge, our study is the first to demonstrate a contrast effect for the perception of beauty in abstract paintings. Our findings suggest that perceptual contrast effects are not necessarily related to semantic content, but can be demonstrated for abstract images as well, i.e., for images that contain no (or only spurious) explicit meaning. This conclusion, however, remains restricted to the group of students with a Western cultural background, who took part in the present study.

The result for cluster 5 shows a pattern opposite to the other 6 clusters in Experiment 1B (Figure 4). In this cluster, the rating was more negative after adaptation to least beautiful images and more positive after adaptation to beauty. The average rating by the participants in cluster 5 was 2.19, which is significantly lower than the mean from all the other clusters (2.81; the closest cluster has an average of 2.62). We tried to elucidate the reason for this unusual evaluation pattern. In particular, we asked whether there was an (inverse) correlation of the cluster-5 rating with respect to the other clusters. We also checked with a linear regression analysis whether the evaluation itself or the magnitude of the adaptation effect of single participants or of the entire cluster 5 correlated with any of the measured statistical image properties. However, none of the image properties analyzed seemed to have had a critical influence on the participants' rating in cluster 5. We cannot exclude the possibility that participants in cluster 5 adapted to some other features (or combinations thereof) or shifted their attention to other criteria than the ones we investigated. A general lack of cooperation or attention cannot be responsible for the inverse contrast effect. In summary, we were not able to clarify why members of cluster 5 differed in their adaptation pattern.

In 2 of the 7 clusters that were used in Experiment 1B, the original rating scores (Experiment 1A) were significantly lower overall, i.e., the paintings were evaluated as less beautiful than in Experiment 1B (*p* < 0.01 and *p* < 0.05, respectively). This difference might possibly be due to a familiarity bias, as suggested by Cutting (2003). In his work on French Impressionism, he proposed that exposure to artworks of a certain style can increase preference for artworks belonging to the same style. For complex images, Berlyne (1970) observed a similar increase of preference that goes along with a decrease of novelty; he found the opposite trend for simple stimuli. Our present findings are in agreement with Berlyne's observation. We would argue that many of the abstract images shown in the experiments were rather complex and may have induced a high level of arousal in most of the participants who were non-experts and thus relatively naïve with regard to abstract art.

To explain the observed perceptual contrast, three possibilities should be considered. First, the effect might be the result of an adaptation to visual beauty. In this scenario, the perception of beauty in an individual observer is modified by changes in the responsiveness of the underlying neural circuits. Second, the observed effect could be the result of a criterion shift, as described by Morgan et al. (2012) in their study on the shift of psychometric sensory discrimination functions. Following this notion, evaluation closely depends on a given criterion. A shift in evaluation may result from viewing a special set of adaptor stimuli (i.e., beautiful rated images) in which the criterion is especially pronounced. Third, conscious or unconscious comparison of the images might have biased the participants' ratings. Cogan et al. (2012) described such a comparison effect on hedonic contrast for stimuli related to face attractiveness. Based on the experimental data, it is not possible to decide which of these mechanisms (or which combination thereof) accounts for the contrast effect observed in the present study. In view of the short presentation times of the images (600 ms), it is likely that perceptual effects play a relatively prominent role in the observed effect compared to cognitive processes. This hypothesis is strengthened by the described correlation between the statistical properties (especially, PHOG self-similarity) and the size of the contrast effect. To discriminate between the role of perceptual vs. cognitive mechanisms in the perception of abstract art would be an interesting aim for future investigations.

### Color features

In Experiment 2, significant correlations were found between beauty ratings and some of the color measures (hue, saturation, and value of the HSV color space; the R and G channels of the RGB color space; and the L and b channels of the Lab color space; see Table 1). These findings are in line with previous studies that revealed a prominent role of color for the aesthetic quality of images. The preference for bright, reddish, and yellowish colors can be explained with the ecological valence theory proposed by Palmer and Schloss (2010) who proposed that color preferences are due to their associations with preferred objects (see Introduction). Unlike the findings by Palmer and Schloss (2010) who showed a general preference for bluish colors, we found a stronger preference for yellowish artworks. However, this difference may well be explained by the different test stimuli used in the experiments (homogeneously colored squares vs. artworks). In another recent investigation, Yanulevskaya et al. (2012) showed that bright and saturated colors generated positive emotions, while darker colors tended to evoke negative emotions. Moreover, Amirshahi et al. (2013) described a strong correlation of beauty ratings with color quantization values and mean color value in a large dataset of figurative paintings. In conclusion, our results confirm the crucial role of color for appreciation of beauty in artworks, which is also evident from the account of this topic in art history and aesthetic theory.

### Statistical properties other than color features

In addition to color values, we measured other statistical properties of images (self-similarity, complexity, anisotropy, Birkhoff-like measure, and aspect ratio), which have been associated with aesthetic judgment, and searched for correlations with beauty ratings. The results of the present study are in line with previous studies that focused on representational art (e.g., Redies et al., 2012; Braun et al., 2013). For example, the mean value for self-similarity obtained in the present study (0.68 ± 0.13 *SD*) is similar to the average value for 197 works of representational art (0.67 ± 0.09 *SD*; Table 1 in Braun et al., 2013). This value comes close to the respective value for photographs of natural scenes (0.64 ± 0.10 *SD*) and is significantly higher than the self-similarity of urban scenes (0.55 ± 0.08 *SD*) or photographs of simple objects (0.54 ± 0.07 *SD*). However, other images like photographs of branches possess an even higher degree of self-similarity (0.77 ± 0.07 *SD*; Braun et al., 2013). It therefore seems possible that there is a degree of self-similarity, for which processing in the visual system is optimized, as proposed by Taylor et al. (2011), and which therefore might evoke an aesthetic response. For our analysis, we used a relatively new approach to measure self-similarity, the PHOG method (see section Experiment 2: Image Analysis). Over all participants, self-similarity values showed no correlation with beauty ratings. However, in some clusters, we obtained positive correlations with beauty, and negative correlations in other clusters. A possible explanation for our findings might be the rather high self-similarity in some of the abstract images, which may have led to some inverse correlations with the beauty rating. Moreover, self-similarity has a significant main effect on clustering (Table 3), indicating that subgroups of persons differ in their preference for self-similarity in abstract paintings.

For complexity and the Birkhoff-like measure, we did not find any significant correlation with beauty ratings. The correlation between beauty appreciation and complexity is thought to be non-linear and to manifest itself in an inverted u-shaped response curve (Berlyne, 1974; Nadal, 2007; Forsythe et al., 2011). Still, even after considering several statistical analyses to account for such an inverted u-shaped response curve, we were not able to detect any correlation. Because complexity is used for calculation of the Birkhoff-like measure, it is not surprising that this measure does not correlate with beauty either.

Also, we did not find any correlations between beauty ratings and anisotropy. Generally, paintings of Western provenance are of low anisotropy (Redies et al., 2012). Our study was the first attempt to search for correlations of anisotropy with subjective ratings on beauty. A causative role of anisotropy in beauty ratings remains to be established. Furthermore, there may be differences between various styles of figurative and abstract art. Finally, in agreement with previous findings (McManus, 1980; Russell, 2000), we found no correlations of beauty ratings with the aspect ratio of the abstract artworks. In summary, next to the color measures, self-similarity seems to be the best predictor for aesthetic appreciation of abstract artworks.

### Differences in beauty preferences between participants

Interestingly, some of these correlations between beauty ratings and image features differed between the subgroups (clusters) of participants (Figure 6). In particular, our results suggest that the participants had individual preferences for specific color combinations and that there were clusters of persons with shared preferences. Correlations were stronger for single clusters than in the analysis over all participants.

We performed the statistical analysis for sets of 3–7 clusters in order to look for consistent clusters. Interestingly, all sets of clusters shared the same group of 3 relatively stable clusters of similar preferences, which are highlighted by lightly colored backgrounds in Figure 6. This correspondence suggests that there are at least 3 subgroups of participants, which differ in their preferences for specific types of beauty in abstract artworks. Other participants, who do not share preferences with these subgroups, may be allocated to the particular group they match best, although they may have a rather singular taste with no preference for a specific combination of the image features studied. Although the clustering was performed exclusively based on subjective evaluations, we found differences of the effect of statistical image properties on the ratings by participants from the different clusters. We therefore propose that the clustering is not accidental but due to preferences for specific image properties by groups of participants (Table 3). Of course, our sample of participants is restricted to students in Germany and may not be representative for the population at large or other cultural backgrounds.

Our findings go along with previous studies that confirmed interindividual differences in aesthetic appraisal. Jacobsen and Höfel (2002) described substantial differences in individuals who evaluated novel graphic patterns with respect to their subjective definition of beauty. Similar to the findings from Experiment 1A, the authors were able to represent individual patterns of judgment more accurately with an individual judgment paradigm compared to a group model (Jacobsen and Höfel, 2002). Interestingly, Vessel and Rubin (2010) assumed that, as a result of shared semantic interpretations, people show a high degree of agreement in appraising the beauty in real-world scenes but a rather individual taste in a non-semantic context, as it is the case for abstract art. Hence, the individual evaluation differences in the present study might partly be explained by the usage of abstract art.

Augustin et al. (2012) focused on word usage for describing art images and obtained evidence for interindividual differences in aesthetic appraisal. While “beautiful” and “ugly” are terms that have a similar meaning for a majority of people, other adjectives for describing art have meanings that are more variable between individuals. This variable usage of words suggests differences in art appreciation between individuals.

We are aware of the limited validity of the clustering results of the present study, as most of the participants in our study shared similar origin, age, and social group. Additionally, due to the experimental design, we presented only a limited selection of artworks. Future studies will have to confirm that clusters with distinct tendencies of preference can also be obtained for other cultures and social groups.

### Final conclusion and outlook

In conclusion, we found a perceptual contrast effect on perceived beauty in abstract art images. Unlike previous adaptation studies on simple stimuli, the present study uses artworks that were rather heterogeneous and some were highly complex. Perhaps not surprisingly, clusters of participants differed in their individual preference for these artworks, and some clusters showed preference for a specific pattern of similar low-level image features present in the artworks. We hypothesize that the perceptual contrast depends, at least in part, on these low-level features, which might be the basis for a criterion shift. However, it was impossible to define a single reason for the perceptual contrast observed in Experiment 1B.

In future studies, it will be of interest to study whether people prefer different image features depending on different styles of art or semantic content. For instance, it can be hypothesized that features that are related to natural depictions may be less important in abstract art whereas, *vice versa*, geometrical shapes and colors may have a stronger influence in abstract art, as they remain the only visual qualities that the observer can refer to. Moreover, in the present study, we focused exclusively on statistical image features that can be processed at low levels of the visual system. Evidently, high-level properties, such as the knowledge about the artist and the artistic style, also play an important role in the aesthetic appreciation of artworks (see, e.g., Leder et al., 2004; Wallraven et al., 2009).

## Author contributions

Gregor U. Hayn-Leichsenring and Christoph Redies designed the study. Gregor U. Hayn-Leichsenring wrote the computer program for the rating experiment. Birgit Mallon carried out the experiments and analyzed the data under Gregor U. Hayn-Leichsenring's supervision. Birgit Mallon, Christoph Redies, and Gregor U. Hayn-Leichsenring produced the figures and wrote the paper.

### Conflict of interest statement

The authors declare that the research was conducted in the absence of any commercial or financial relationships that could be construed as a potential conflict of interest.
