# Supplementary material for: Beauty in abstract paintings: perceptual contrast and statistical properties
Source: Front Hum Neurosci. 2014 Mar 21;8:161. doi: 10.3389/fnhum.2014.00161 (PMC3968763; doi:10.3389/fnhum.2014.00161)
Supplement: Supplementary file 1 [file Presentation1.PDF]

Appendix A

List of artworks used in the present study. The numbers in square brackets indicate the number of paintings or drawings that were analyzed for each artist.

Bargheer, Eduard [1]: Ischia
Baumeister, Willi [1]: Aru mit Gelb
Birolli, Renato [1]: Green and blue over Liguria
Brust, Karl F. [1]: Geheimnisvolles Blau
Cavael, Rolf [1]: Komposition
de Kooning, Willem [5]: Untitled (Summer in springs), 1962; Montauk II, 1969; Untitled VIII, 1980; Pirate (Untitled II), 1979; Untitled IV
de Stael, Nicolas [1]: Landscape, Honfleur
Dexel, Walter [5]: Sign; With two Crosses; Painting; Stijl 4; Composition, 1927
Diebenkorn, Richard [1]: Urbana No. 6
Dubuffet, Jean [2]: Table de barbe, 1959; Joie de terre, 1959
Fontana, Lucio [2]: Anticipation; Spatial concept
Förg, Günther [1]: Untitled (fg 00035), 1990
Francis, Sam [5]: Paris, Summer; Untited, 1954-56; Study for Moby Dick, 1958; Kekek korul; Untitled, 1957
Frankenthaler, Helen [6]: Tribal Sign; Tiger`s Eye; Spring Blizzard, 1957; Seven Types of Ambiguity; Monotype XXIX; Madame de Pompadour
Gorky, Arshile [1]: Waterfall
Gottlieb, Adolph [1]: Nadir, 1952
Guston, Philip [1]: For M
Hesse, Eva [5]: Accession; Untitled, 1969; Untitled (Two Circles), 1968; Untitled, 1963; Untitled, 1968
Hoehme, Gerhard [5]: Das wilde blaue Band, 1956/57; Paramnesis; Netim; Das Klingelbild, 1965; Title unknown to the authors
Hofmann, Hans [5]: Symphony; House in Storm; Exorbitanz, 1949; Untitled, not dated; Red Bird, 1951
Imkamp, Wilhelm [1]: Abstrakte Komposition
Johns, Jasper [2]: Target; 0 through 9
Klee, Paul [5]: Farbtafel (auf maiorem Grau), 1930; Haus, aussen und innen, 1930; Alter Klang, 1925; Rhythmisches, strenger und freier, 1930; Monument im Fruchtland, 1929
Klein, Yves [5]: Untitled (IKB 93), 1961; Untitled (M 38), 1955; Untitled (M 35), 1957; Untitled (M 47), 1957; Shower from mars (COS 34)
Kleint, Boris [1]: Ferne Welten
Kline, Franz [1]: Untitled, 1957
Kljun, Iwan [1]: Suprematism, 1915/16
Krasner, Lee [5]: Cornucopia, 1958; Courtship, 1966; Listen, 1957; Streched Yellow; Polar Stampede, 1961
Levedag, Fritz [1]: Komposition in blau
Louis, Morris [3]: Dalet Nun; Omega IV; Untitled, 1956
Malevich, Kasimir [6]: Black Cross, 1923; Black Square, 1923; Suprematism, 1915; Suprematism, 1921-1927; Suprematism. Painterly realism of a Footballer, 1915; Black circle
Manessier, Alfred [1]: La Nuit de Gethsemane, 1952
Matjuschin, Michail [1]: Movement in Space, 1917/18
Meistermann, Georg [1]: Luftstreit
Mitchell, Joan [1]: Untitled, 1960; Hemlock
Moholy Nagy, László [5]: Red collage, 1921; Composition, 1923; Composition Q XX, 1923; A m

4, 1926; Yellow circle
Mondrian, Piet [5]: Composition with yellow and blue, 1932; Composition, 1914; Composition, 1916; Composition, 1920; Composition with lines, 1917
Motherwell, Robert [5]: Lyric Suite; Medusa Series; Night Music Opus No. 7; Drawing No. 10; Wall painting No. III, 1953
Müller-Hufschmid, Willi [1]: Der weiße Punkt
Nay, Ernst Wilhelm [5]: Untitled, 1966; Gelb, 1967; Strahlung, 1964; Mit aphoristischem Rot, 1954; Title unknown to the authors
Newman, Barnett [2]: White fire I; Onement I
Pollock, Jackson [5]: Number 4, 1950; Untitled, 1946; Number 48, 1949; Gothic, 1944; Reflection of the big dipper, 1947
Popowa, Ljubow [4]: Composition, 1916; Space-Power-Construction; Pictorial architecture. Black, Red, Grey; Pictorial architecture, 1916
Rauschenberg, Robert [1]: Memorandum on Bids, 1956
Reinhardt, Ad [1]: Yellow Painting, 1949
Richter, Gerhard [5]: Abstraktes Bild, 1977; Abstraktes Bild, 1979; Abstraktes Bild, 1981; 4096 Colours, 1974; Red-Blue-Yellow
Rodtschenko, Alexander M. [1]: Black on black
Rosanova, Olga [2]: Suprematistic composition, 1916; Non-Representational Composition, 1917
Rothko, Mark [5]: No. 20, 1949; White and brick on light red, 1954; Untitled, 1946; Magenta, black, green on orange, 1949; Untitled, 1969
Smith, David [2]: Untitled, 1957; Untitled, 1958
Springer, Ferdinand [1]: Nachtblume
Stamos, Theodoros [1]: Documenta II
Still, Clyfford [1]: 1949 H
Tàpies, Antoni [1]: Painting with red cross, 1954
Trökes, Heinz [1]: Gefährdete Insel
Tschaschnik, Ilja [1]: Suprematism 1922/23
Twombly, Cy [1]: Nini's painting, 1971
Vieira da Silva, Maria H. [1]: Composition
Werner, Theodor [1]: Ausstrahlende Flecken
Winter, Fritz [1]: Wandlung
Wols [4]: Ockerfarbener Grund, bespritzt mit Schwarz; Ohne Titel (Bäume); Malerei, 1946/47; Untitled, 1947
